# Incidence, causes, and maternofetal outcomes of obstructed labor in Ethiopia: systematic review and meta-analysis

**DOI:** 10.1186/s12978-021-01103-0

**Published:** 2021-03-10

**Authors:** Asteray Assmie Ayenew

**Affiliations:** Midwifery Department, College of Medicine and Health Sciences, Bahir Dar University, Bahir Dar, Ethiopia

**Keywords:** Obstructed labor, Obstetric complications, Maternal mortality and morbidity, Systematic review and meta-analysis, Ethiopia

## Abstract

**Background:**

Obstructed labor is a preventable obstetric complication. However, it is an important cause of maternal mortality and morbidity and of adverse outcomes for newborns in resource-limited countries in which undernutrition is common resulting in a small pelvis in which there is no easy access to functioning health facilities with a capacity to carry out operative deliveries. Therefore, this systematic review and meta-analysis aimed to estimate the incidence, causes, and maternofetal outcomes of obstructed labor among mothers who gave birth in Ethiopia.

**Method:**

for this review, we used the standard PRISMA checklist guideline. Different online databases were used for the review: PubMed, Google Scholar, EMBASE, Cochrane Library, HINARI, AFRO Library Databases, and African Online Journals. Based on the adapted PICO principles, different search terms were applied to achieve and access the essential articles. The search included all published and unpublished observational studies written only in the English language and conducted in Ethiopia. Microsoft Excel 16 was used for data entrance, and Stata version 11.0 (Stata Corporation, College Station, Texas, USA) was used for data analysis.

**Results:**

I included sixteen (16) primary studies with twenty-eight thousand five hundred ninety-one (28,591) mothers who gave birth in Ethiopia. The pooled incidence of obstructed labor in Ethiopia was 12.93% (95% CI: 10.44–15.42, I^2^ = 98.0%, *p* < 0.001). Out of these, 67.3% (95% CI: 33.32–101.28) did not have antenatal care follow-up, 77.86% (95% CI: 63.07–92.66) were from the rural area, and 58.52% (95% CI: 35.73– 82.31) were referred from health centers and visited hospitals after 12 h of labor. The major causes of obstructed labor were cephalo-pelvic disproportion 64.65% (95% CI: 57.15– 72.14), and malpresentation and malposition in 27.24% (95% CI: 22.05–32.42) of the cases. The commonest complications were sepsis in 38.59% (95% CI: 25.49–51.68), stillbirth in 38.08% (95% CI: 29.55–46.61), postpartum hemorrhage in 33.54% (95% CI:12.06– 55.02), uterine rupture in 29.84% (95% CI: 21.09–38.58), and maternal death in 17.27% (95% CI: 13.47–48.02) of mothers who gave birth in Ethiopia.

**Conclusion:**

This systematic review and meta-analysis showed that the incidence of obstructed labor was high in Ethiopia. Not having antenatal care follow-up, rural residency, and visiting hospitals after 12 h of labor increased the incidence of obstructed labor. The major causes of obstructed labor were cephalo-pelvic disproportion, and malpresentation and malpresentation. Additionally, the commonest complications were sepsis, stillbirth, postpartum hemorrhage, uterine rupture, and maternal death. Thus, promoting antenatal care service utilization, a good referral system, and availing comprehensive obstetric care in nearby health institutions are recommended to prevent the incidence of obstructed labor and its complications.

**Supplementary Information:**

The online version contains supplementary material available at 10.1186/s12978-021-01103-0.

## Plain english summary

Labor is considered obstructed when the presenting part of the fetus cannot progress into the birth canal, despite strong uterine contractions. The most frequent cause of obstructed labor is cephalo- pelvic disproportion, a mismatch between the fetal head and the mother's pelvic brim. The fetus may be large to the maternal pelvic brim, such as the fetus of a diabetic woman, or the pelvis may be contracted, which is more common when malnutrition is prevalent. Some other causes of obstructed labor may be malpresentation and malposition of the fetus (shoulder, brow, or occipito- posterior positions). In rare cases, locked twins or pelvic tumors can cause obstruction. To the best of my knowledge, no systematic review was conducted to estimate the national prevalence of obstructed labor. Therefore, this systematic review and meta-analysis aimed to estimate the incidence, causes, and maternofetal outcomes of obstructed labor among mothers who gave birth in Ethiopia. Sixteen (16) primary studies with twenty-eight thousand five hundred ninety-one (28,591) mothers who gave birth in Ethiopia were included. The pooled incidence of obstructed labor) mothers who gave birth in Ethiopia was 12.93%. Out of these, 67.3% did not have antenatal care follow-up, 77.86% were from the rural range, and 58.52% were referred from health centers and visited hospitals after at least 12 h of labor. The major causes of obstructed labor were cephalo-pelvic disproportion 64.65%, and malpresentation and malposition in 27.24% of the cases. The commonest complications were sepsis in 38.59%, stillbirth in 38.08%, postpartum hemorrhage in 33.54%, uterine rupture in 29.84%, and maternal death in 17.27% of mothers who gave birth in Ethiopia. Thus, promoting antenatal care service utilization, a good referral system, and availing comprehensive obstetric care in nearby health institutions are recommended to prevent the incidence of obstructed labor and its complications.

## Introduction

Obstructed labor is defined as a failure of the fetal presenting part to descent in the birth canal due to mechanical reasons, despite having adequate uterine contraction [1, 2]. It is diagnosed when the duration of labor is prolonged, a laboring mother became unable to support herself or unable to move her lower extremities, with deranged vital signs, distended bladder, Bandle’s ring formed in the lower uterine segment, fetal distress or death, edematous vulva, big caput, significant molding, foul-smelling and thick meconium-stained amniotic fluid [3]. Neglected obstructed labor (OL) is a major cause of both maternal and newborn morbidity and mortality. The obstruction can only be alleviated by means of operative delivery, either cesarean section or other instrumental delivery (forceps, vacuum extraction, or simphysiotomy) [4].

Globally, at least 585,000 women die each year from complications of pregnancy and childbirth. More than 70% of all maternal death is due to five major complications: hemorrhage, infection, unsafe abortion, hypertensive disorders of pregnancy, and obstructed labor [5]. Among these etiologies, obstructed labor is one of the most common causes of maternal illness and death in sub-Saharan Africa and Southeast Asia. Worldwide, obstructed labor occurs in an estimated 5% of pregnancies and accounts for an estimated 8% of maternal deaths. The majority of the maternal deaths occurred in the poor, illiterate, hard-to-reach women who are living in rural areas with limited or no access to skilled birth attendants [6].

It is an indicator of inadequacy and poor quality of obstetric care, and immediate cause of maternal and prenatal morbidity and mortality due to uterine rupture, complications of cesarean delivery, postpartum hemorrhage, anesthesia complications, puerperal sepsis, asphyxia, and brain damage. Moreover, neglected obstructed labor resulted from poverty and prohibiting high cost of maternal care in hospitals, ignorance, illiteracy, obstructed transportation, socio-cultural belief to achieve vaginal delivery at all cost, late referrals, and aversion to caesarean delivery and hospital delivery especially after a previous caesarean operation [7].

The fetus dies first, followed by the death of the mother that puts the lives of other children in the family in jeopardy. Many parturient women die undelivered and delivered by postmortem cesarean delivery [8]. The few women with intra uterine infection and fetal deaths that managed to reach the hospital alive, the tip of the iceberg, were usually delivered by cesarean operations because of lack of the skills to perform the simpler fetal destructive vaginal operations, and this is associated with the gamut of complications [5, 9]. The risk of maternal death after abdominal delivery in such a septic condition can be very high [10].

Other complications of abdominal delivery include sepsis and septic shock, anemia, blood transfusion, wound infection, and burst abdomen, prolonged hospital stay, high cost of care, infertility, aversion to hospital delivery, and caesarean delivery in a subsequent pregnancy, obstetric fistulas, abandonment, and even divorce. Complications that have been attributed directly to fetal destructive vaginal operations include uterine rupture in 2.6–9.1% of cases, postpartum hemorrhage in 4.5%, and cervical and vaginal lacerations in 1.3% [11].

Maternal mortality arising from destructive operations in the management of neglected obstructed labor ranged from 0 to 2.7% when compared to 7.5% for abdominal delivery [11, 12]. Certainly, fetal destructive operation is safer than abdominal delivery in neglected obstructed labor with fetal demise provided the uterus has not ruptured and is not at the verge of rupture.

Maternal and perinatal mortality and morbidity associated with obstructed labor are almost totally prevented in developed countries because of improved nutritional status, wide health coverage, adequate transportation and communication system, availability of trained health personnel, optimal antenatal and intrapartum care, and other related factors [13].

In most sub-Saharan countries including Ethiopia, women are traditionally expected to give birth at home and consequently delay their health care seeking in childbirth, even if complications arise. Moreover, women are often marginalized in decision making regarding where and when to seek care [14]. Unofficial financial demands from health workers prevent women from badly needed maternal health services. Inadequately developed health care systems including poor infrastructure, poor transportation and poor obstetric services are also major contributors to obstructed labor [15].

Obstructed labor has different magnitudes in different developing countries ranging from 2 to 8%. When we come to Africa some research finding showed that the magnitude of obstructed labor was more than the above determined once; In Uganda and Ethiopia, the magnitude of obstructed labor was described as 10.5% and 12.2% respectively [2, 16]. In Ethiopia, despite different strategies to reduce morbidities and mortalities, among the 412 maternal deaths per 100,000 live births annually, 19.1% happened due to obstructed labor [17, 18].

Apart from maternal deaths, obstructed labor had different maternal outcomes such as uterine rupture, postpartum hemorrhage, puerperal sepsis, bladder injury, Vesico-Vaginal fistula (VVF), recto-vaginal fistula (RVF), and fetal outcomes including birth asphyxia, stillbirth, neonatal jaundice, and umbilical sepsis [3, 19, 20]. By far, the most severe and distressing long-term condition following obstructed labor is obstetric fistula which causes serious social issues of divorce, separation from religious exercises, detachment from their families which can worsen poverty, and malnutrition [20]. Despite these severe complications, the prevalence of obstructed labor is still high in Ethiopia ranging from 3.3% in Tigray region [21] to 34.3% in Oromia region [22]. Therefore, the aim of this systematic review and meta-analysis was to estimate incidence, causes, and maternofetal outcomes of obstructed labor among mothers who gave birth in Ethiopia.

## Methods

This systematic review and meta-analysis were conducted to estimate incidence, causes, and maternofetal outcomes of obstructed labor among mothers who gave birth in Ethiopia. We used the Preferred Reporting Items for Systematic Reviews and Meta-Analyses (PRISMA) checklist guideline [23] (Additional file 1).

### Searching strategy

First, the PROSPERO database and database of abstracts of reviews of effects (DARE) (http://www.library.UCSF.edu) were searched to check whether published or ongoing projects exist related to the topic. The literature search strategy, selection of studies, data extraction, and result reporting were done in accordance with the Preferred Reporting Items for Systematic Reviews and Meta-Analyses (PRISMA) guidelines [24]. We searched PubMed, Google Scholar, EMBASE, Cochrane Library, HINARI, AFRO Library Databases, and African Online Journal databases for all available studies using the following terms: "obstructed labor", "prolonged labor", "obstetric complications", "childbirth", "labor abnormalities", "factors", and "Ethiopia". The search string was developed using "AND" and "OR" Boolean operators. Searching terms were based on adapted PICO principles to search through the above-listed databases to access all relevant articles. For unpublished studies, the official website of Ethiopian's University research repository online library (University of Gondar and Addis Ababa University) were used. The searching period was from September 1/2020 to November 30/2020.

### Inclusion and exclusion criteria

All observational studies reporting the incidence of obstructed labor and/or associated factors in Ethiopia were included in this review. Both unpublished and published research articles, conducted in English language were included. Whereas duplicated studies, case reports, qualitative studies, anonymous reports, articles without full text, and abstract and editorial reports were excluded from the study.

### Operational definition

Obstructed Labor: also known as labor dystocia, is a failure to progress due to mechanical problems—a mismatch between fetal size, or more accurately, the size of the presenting part of the fetus, and the mother’s pelvis, although some malpresentation, notably a brow presentation or a shoulder presentation. it is diagnosed when the duration of labor > 24 h, a laboring mother became unable to support herself or unable to move her lower extremities, with deranged vital signs, distended bladder, Bandle’s ring formed in the lower uterine segment, fetal distress or death, edematous vulva, big caput, significant molding, foul-smelling and thick meconium-stained amniotic fluid [3, 25].

#### Causes of obstructed labor

The commonest cause of obstructed labor is craniopelvic disproportion (CPD). This could arise as a result of reduced pelvic dimension from childhood, maternal malnutrition, infection, poliomyelitis, deformity, sickle cell disease, or in teenagers increased diameter of the presenting part, such as malposition and malpresention. These include brow presentation, compound presentation, occipto-posterior, and mento-posterior in face presentation and congenital malformation (hydrocephalus, fetal ascites, and double monsters) [26, 27].

#### Complications of obstructed labor

Apart from maternal death, obstructed labor had different maternal outcomes such as uterine rupture, postpartum hemorrhage, puerperal sepsis, Vesico-Vaginal fistula (VVF), recto-vaginal fistula (RVF), and fetal outcomes including birth asphyxia, stillbirth, neonatal jaundice, and umbilical sepsis. Women who experience obstructed labor for a prolonged time can be complicated with fistulas. Besides their physical wounds, serious social issues of divorce, separation from religious exercises, detachment from their families which can worsen poverty, and malnutrition are the major problems of obstructed labor [3, 19, 20].

#### Cephalopelvic disproportion (CPD)

Is an inadequate size of the maternal pelvis, compared to the fetal head, which prevents the fetus from passing through the pelvic cavity during delivery, and causes obstructed labor [28].

### Quality assessment

After collecting the findings from all databases, the articles were exported to Microsoft Excel spreadsheet. The methodological quality of each study (sampling strategy, response rate, and representativeness of the study), comparability, and outcome were checked using the NOS tool. Newcastle–Ottawa Quality Assessment Scale (NOS) for cross-sectional, and case–control studies was used to assess the methodological quality of a study, and to determine the extent to which a study has addressed the possibility of bias in its design, conduct, and analysis [29]. All included articles scored (NOS) 7 and more can be considered as “good” studies with low risk (Additional file 2).

### Data extraction

Microsoft Excel (2016), and Stata version 11.0 (Stata Corporation, College Station, Texas, USA) software were used for data entry and analysis, respectively. The data was extracted by using a standardized Joanna Briggs Institute (JBI) data extraction format. During data extraction; the name of the author, sample size, publication year, study design, prevalence, response rate, population outcome, study site, and different contributing factors were included. Moreover, the incidence, and outcomes of obstructed labor with 95% CI and associated factors were collected [30].

### Statistical analysis

As the test statistic showed significant heterogeneity among studies (I^2^ = 98.0%, *p* < 0.05) the Random-effects model was used to estimate the DerSimonian and Laird's pooled effect [31]. Cochran’s Q chi-square statistics and I^2^ statistical test was conducted to assess the random variations between primary studies [32]. In this study, the heterogeneity of included studies was interpreted as an I^2^ value of 25% = low, 50% = moderate, and 75% = high [33]. In case of high heterogeneity, subgroup analysis and sensitivity analyses were run to identify possible moderators of this heterogeneity. Potential publication bias was assessed by visually inspecting funnel plots and objectively using the Egger’s test (i.e. *p* < 0.05) [34]. To account for any publication bias, we used the trim-and-fill method, based on the assumption that the effect sizes of all studies are normally distributed around the center of a funnel plot. The meta-analysis was performed using the Stata version 11.0 (Stata Corporation, College Station, Texas, USA) software. Finally, for all analyses, *P* < 0.05 was considered statistically significant.

## Results

### Study selection and data extraction

**Fig. 1:**
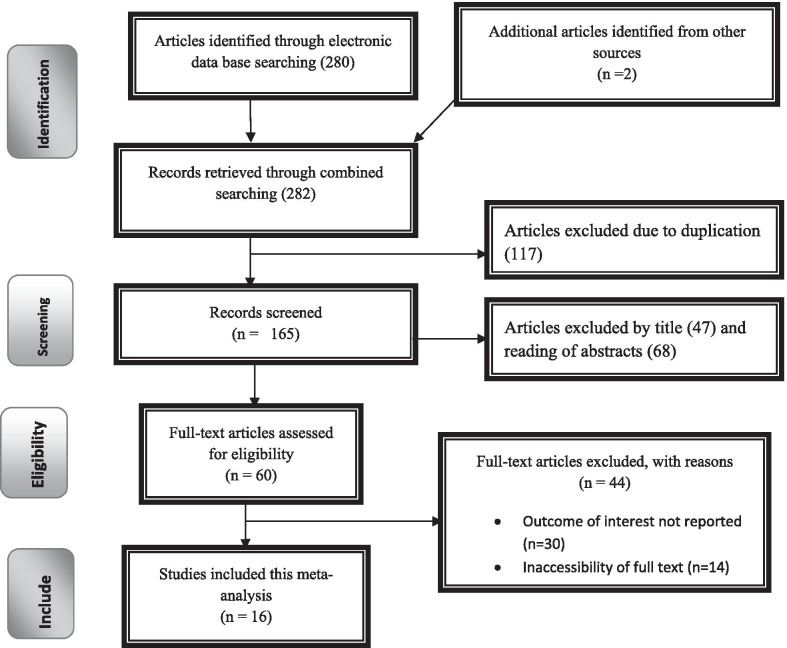
Flow chart of study selection for systematic review and meta-analysis of obstructed labor among mothers who gave birth in Ethiopia

The search strategy identified 80 articles from PubMed, 60 articles from Google Scholar, 45 articles from Cochrane Library, 10 articles from African Journals Online, 7 articles from Ethiopian’s University online library, and 2 articles from manual search. Of which, 134 were excluded due to duplication, 35 through review of titles and abstracts. Additionally, 44 full-text articles were excluded for not reporting the outcome variable and other reasons. Finally, 16 articles were included to analyze the incidence, outcome, and associated factors of obstructed labor (Fig. 1).

### Study characteristics

In this review, 16 relevant studies were included with a sample size of 28,951. Among sixteen studies thirteen were cross sectional [1, 14, 35–45] and three case-controls [46–48] in study design. Regarding the geographical area, six from Oromia [14, 35, 36, 40, 41, 47], four [3, 37, 39, 42] from Southern Nation Nationalities and People (SNNPR), and four [1, 38, 43, 48] from Tigray region, two studies [45, 46] were from Amhara region. Among the included studies the largest sample size was 13,425 [41], whereas the smallest was 90 [48] (Table 1).

**Table 1:** Descriptive summary of nineteen included studies in the systematic review and meta-analysis

Authors	Sample size	Prevalence (%)	Study region	Study design	complications of obstructed labor
Shimelis et al. [35]	1468	12.2	Oromia	Cross sectional	Uterine rupture, sepsis, and low first minute APGAR score
Andualem et al. [37]	1825	7.95	SNNPR	Cross sectional	postpartum hemorrhage, uterine rupture, and perinatal mortality
Amanuel et al. [21]	5980	3.3	Tigray	Cross sectional	Caesarean section, craniotomy, instrumental delivery, hysterectomy, ruptured uterus, Maternal mortality
Ashebir et al. [41]	13,425	7	Oromia	Cross sectional	Ruptured uterus, stillbirth, maternal mortality, and perinatal death
Mulugeta et al. [46]	801	N/A	Amhara	Case control	Cesarean section, infertility, and uterine rupture
Tizita et al. [22]	385	34.3	Oromia	Cross sectional	Maternal death, uterine rupture, hysterectomy, and anemia
Sisay et al. [39]	327	15.6	SNNPR	Cross sectional	Ruptured uterus, cesarean section, wound infection, and neonatal death
Yemane et al. [38]	1231	14.7	Tigray	Case control	Stillbirth, perinatal mortality, and neonatal mortality
Tewodros et al. [48]	90	N/A	Tigray	Cross sectional	Sepsis, postpartum hemorrhage, Vesico Vaginal Fistula, anemia, stillbirth, birth asphyxia, and birth injury
Daniel et al. [14]	321	18.1	Oromia	Cross sectional	postpartum hemorrhage, ruptured uterus, puerperal sepsis, and maternal death
Ritbano et al. [42]	344	18.6	SNNPR	Case control	Maternal death, anemia, and infection
Johannes et al. [47]	143	N/A	Oromia	Cross sectional	sepsis, hemorrhage that required transfusion of several units of blood, ruptured uterus, respiratory tract infection with cardiac failure), low APGAR score, and immediate newborn death
Asnakech et al. [36]	384	9.6	Oromia	Cross sectional	N/A
Gebresilasea et al. [43]	616	6.7	Tigray	Cross sectional	stillbirths or had died immediately after delivery, Postpartum hemorrhaged, puerperal sepsis, uterine rupture, hysterectomy, and cesarean section
Wayu et al. [44]	844	16.8	SNNPR	Cross sectional	Intrapartal fetal and early neonatal deaths, instrumental delivery, and meconium stained amniotic fluid
Oumar et al. [45]	407	10.7	Amhara	Cross sectional	Maternal death, anemia, stillbirth, and hysteretomy

### Incidence of obstructed labor in Ethiopia

**Fig. 2:**
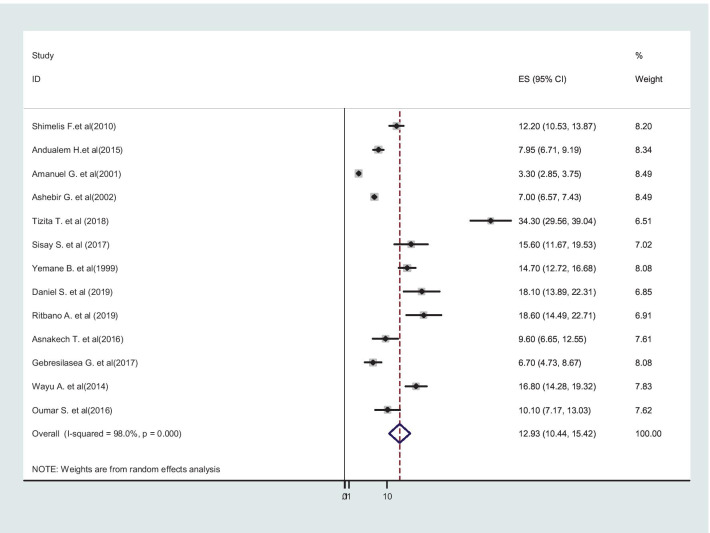
Forest Plot for the pooled incidence of obstructed labor among mothers who gave birth in Ethiopia, 2020

Primarily, all three case–control [46–48] studies were not considered in the incidence estimation, because they did not report the incidence of obstructed labor, but all studies were included in factor analysis. The pooled incidence of obstructed labor is presented on a forest plot (Fig. 2). Therefore, the estimated incidence of obstructed labor among mothers who gave birth in Ethiopia was 12.93% (95% CI: 10.44**–**15.42, I^2^ = 98.0%, *p* < 0.001).

### Publication bias

**Fig. 3:**
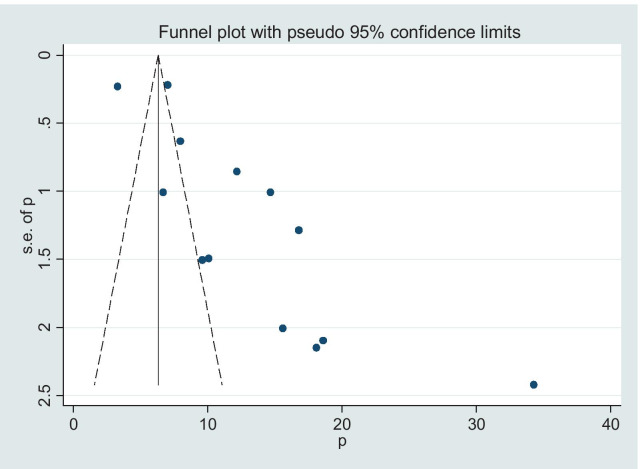
Funnel plot with 95% confidence limits of the pooled incidence of obstructed labor among mothers who gave birth in Ethiopia, 2020

The funnel plot was assessed for asymmetry distribution of the prevalence of obstructed labor among mothers who gave birth in Ethiopia (Fig. 3). Egger's regression test showed a p-value of 0.259 with no evidence of publication bias.

### Sensitivity analysis

**Fig. 4:**
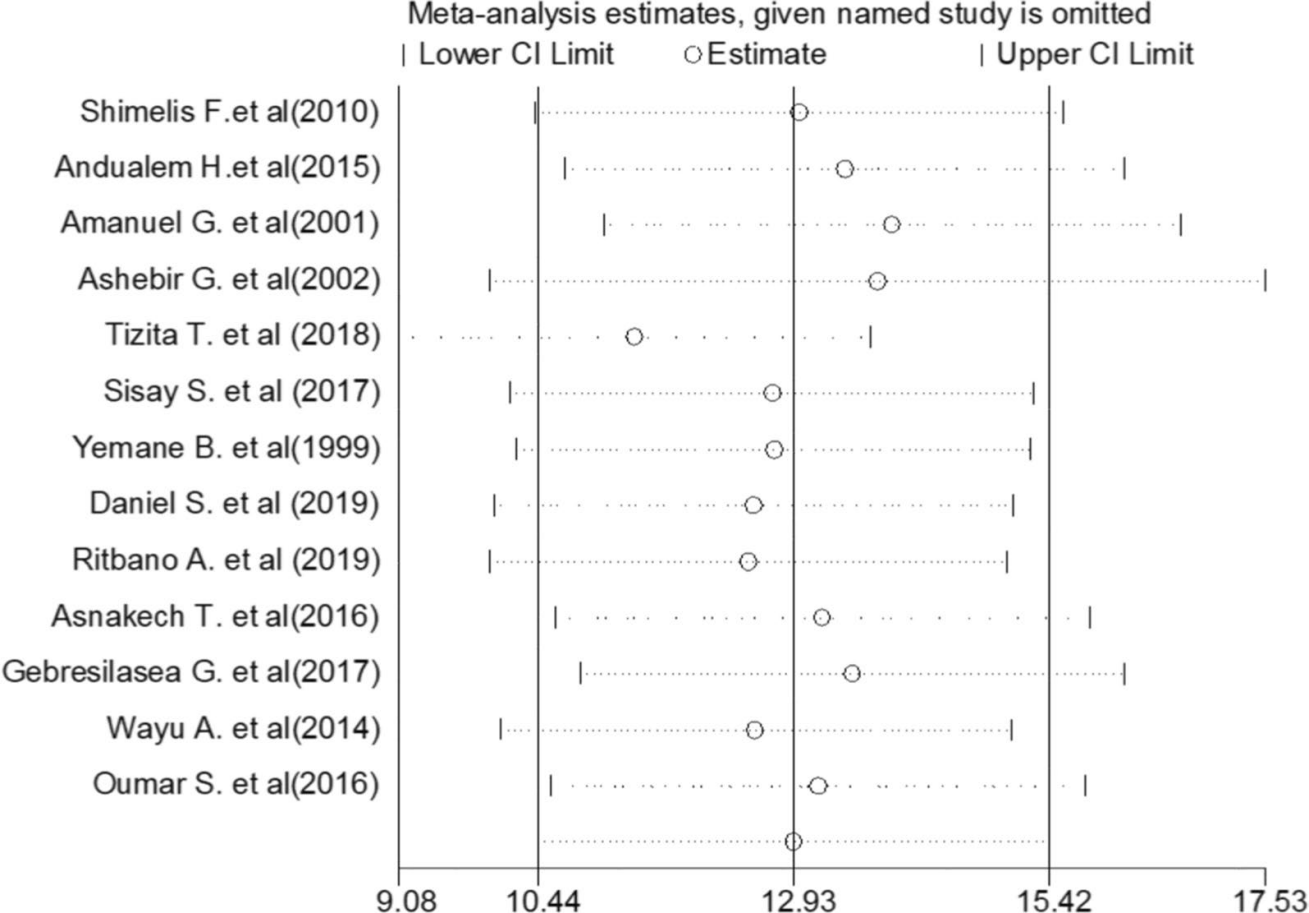
Sensitivity analysis of the pooled incidence of obstructed labor among mothers who gave birth in Ethiopia, 2020

This systematic review and meta-analysis showed that the point estimate of its omitted analysis lies within the confidence interval of the combined analysis. Therefore, trim and fill analysis was no further computed (Fig. 4).

### Subgroup analysis

**Fig. 5:**
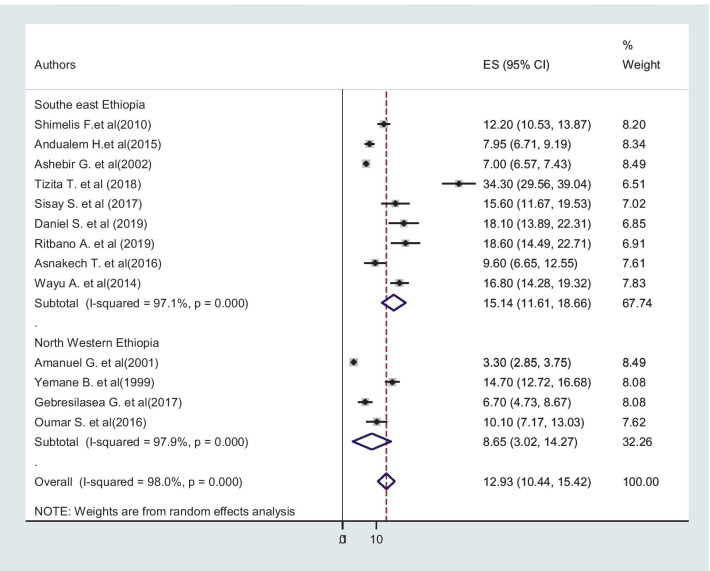
Subgroup analysis of the pooled incidence of obstructed labor among mothers who gave birth in Ethiopia based on the study region, Southeastern Ethiopia

**Fig. 6:**
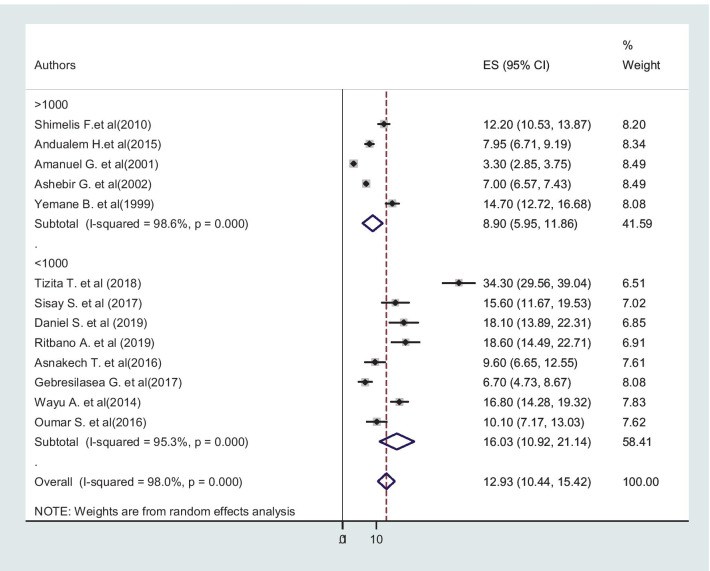
Subgroup analysis of the pooled incidence of obstructed labor among mothers who gave birth in Ethiopia based on sample size

Subgroup analysis was employed with the evidence of heterogeneity. In this study, the Cochrane I^2^ statistic was 98.0%, *P* < 0.001, shower the presence of marked heterogeneity. Therefore, subgroup analysis was done using the study region and sample size. As a result, obstructed labor was high in Southeastern Ethiopia 15.14% (95% CI: 11.61–18.66), regarding sample size, the highest incidence was in the study with the sample size less than 1000 [16.93% (95% CI: 10.92–21.14)] (Figs. 5 and 6).

### Risk factors for the incidence of obstructed labor

The association between not having antenatal care follow-up, rural residency, referred from health centers and visited hospitals after at least 12 h of labor with obstructed labor was carried out.

A total of six articles were included to identify the association between referred from health centers and visited hospitals after at least 12 h of labor and obstructed labor. Mother’s referred from health centers, and visited hospitals after at least 12 h of labor develop obstructed labor by 58.52% than mothers who visited hospitals in short hours of labor (58.52%, 95% CI: 35.73–82.31).

A total of five articles were included to identify the association between rural residency and obstructed labor. Mother’s residency (as defined as rural and urban) was significantly associated with obstructed labor. Mother’s from rural areas were more likely to have obstructed labor than those (women) from urban areas, 77.86% (95% CI: 63.07–92.66).

Moreover, four studies showed a significant association between not having antenatal care follow-up and obstructed labor. Mother’s who did not have antenatal care follow-up were 67.3% more likely to develop obstructed labor (67.3%, 95% CI: 33.32–101.28) compared to mothers who had antenatal care follow-up (Table 2).

**Table 2:** Factors for the incidence of obstructed labor in Ethiopia

Factors for obstructed labor	Number of studies	Model	Status of heterogeneity	Prevalence (95% CI)	I^2^ (%)	*P*-value
Rural residency	5	Random	Marked	77.86% (95% CI: 63.07–92.66)	99.6	≤ 0.001
Not having ANC	4	Random	Marked	67.3% (95% CI: 33.32–101.28)	99.8	≤ 0.001
Referred from health center after 12 h duration of labor	6	Random	Marked	58.52% (95% CI: 35.73– 82.31)	98.1	≤ 0.001

**Fig. 7:**
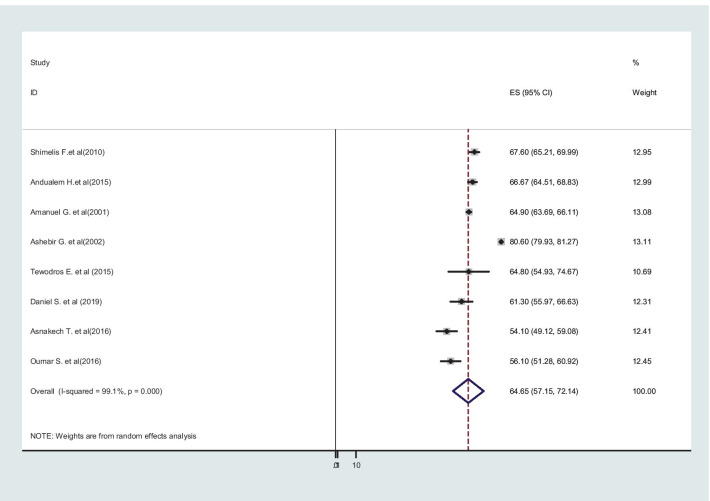
Cephalo-pelvic disproportion as a major cause of obstructed labor among mothers who gave birth in Ethiopia

**Fig. 8:**
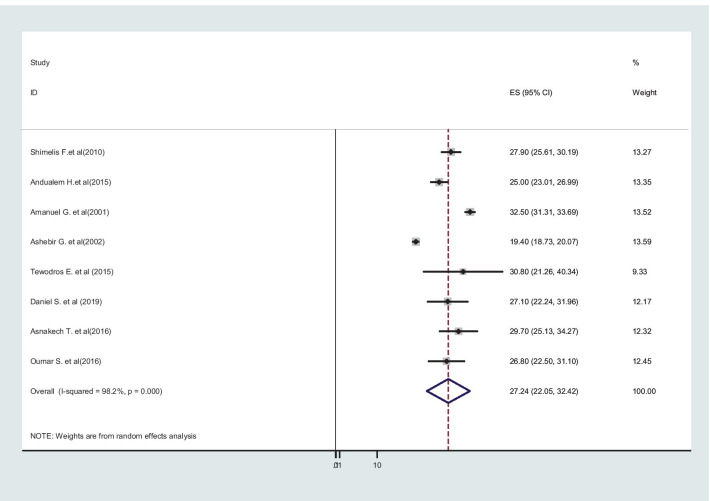
Malpresentation and malposition as a major cause of obstructed labor among mothers who gave birth in Ethiopia

Additionally, the two major causes of obstructed labor were cephalo-pelvic disproportion 64.65% (95% CI: 57.15–72.14), and malpresentation and malposition in 27.24% (95% CI: 22.05–32.42) of the cases (Figs. 7 and 8).

Materno-fetal complications following obstructed labor in Ethiopia.

Following obstructed labor, different adverse maternal and neonatal complications were reported. Sepsis, stillbirth, postpartum hemorrhage, uterine rupture, and maternal death were the most common complications following obstructed labor (Table 3**).**

**Table 3:** Materno-fetal complications following obstructed labor in Ethiopia

Complication s of obstructed labor	Number of included studies	Model	Status of heterogeneity	Prevalence (95% CI)	I^2^ (%)	*P*-value
Sepsis	5	Random	Marked	38.08% (95% CI: 29.55– 46.61)	97.5	≤ 0.001
Stillbirth	8	Random	Marked	38.59% (95% CI: 25.49– 51.68)	99.8	≤ 0.001
Postpartum hemorrhage	5	Random	Marked	33.54% (95% CI: 12.06– 55.02)	99.2	≤ 0.001
Uterine rupture	10	Random	Marked	29.84% (95% CI: 21.09– 38.58)	98.7	≤ 0.001
Maternal death	6	Random	Marked	17.27% (95% CI: 13.47– 48.02)	97.2	≤ 0.001

## Discussion

Obstructed labor is a life-threatening obstetrical complication associated with significant maternal as well as fetal morbidity and mortality. Early recognition and immediate intervention are important to prevent associated complications and to improve maternal and fetal outcomes [21]. Several interventions, such as the utilization of the partograph to monitor labor and provision of emergency obstetrical care services have been proposed to reduce the incidence of obstructed labor, and its squeal. However, the prevalence remains high in the developing countries [49].

The purpose of this review was to assess the incidence, mernofetal outcome, and associated factors of obstructed labor by reviewing the findings of available primary studies. The pooled incidence of obstructed labor in Ethiopia was 12.93%. The result higher than the studies conducted in India [50] 1.9%, Pakistan [51] 2.1%, Nigeria [52] 4.7%, and Uganda 10.5% [53]. The possible reason might be poor ANC follow up, high homebirth prevalence, teenage pregnancy, low socioeconomic status, poor infrastructure, and poor referral system in Ethiopia [54–56].

This study also elucidated that, 67% of the obstructed labor cases did not have ANC follow-up during pregnancy. The result is supported by studies conducted in Pakistan [57], and Nigeria [20]. This might be the fact that not having antenatal care during pregnancy may decrease women knowledge about their pregnancy condition like multiple pregnancies, big baby, fetal anomalies, and other risk factors for obstructed labor. Moreover, women who don’t have antenatal care are prone to home childbirth, poor awareness about birth preparedness and complication readiness plan, and danger signs of pregnancy which in turn increase the risk of obstructed labor.

Among mothers who had obstructed labor, 77.86% were from rural areas. The result is in line with studies conducted in Uganda [53] and Bangladesh [57]. This could be due to women residing in rural areas, health facilities are distant, and accesses to information about institutional deliveries are limited. This might result in a delay to decide for seeking health care as early as possible and delay in reaching a health facility which contributes a lot to the occurrence of obstructed labor.

Additionally, 58.52% of mothers who had obstructed labor were referred from health centers and visited hospitals after at least 12 h of labor. The result is supported by studies conducted In Ghana [58] and Eastern Uganda [59]. This could be explained as women might be referred after a long time of stay at the lower level facilities either due to lack of transportation, lack of infrastructure, poor decision of health care providers, and refusal of families which promote the occurrence of obstructed labor.

The main obstetric causes of obstructed labor in this review were cephalopelvic disproportion accounted for 64.65%. The result is supported by studies in Uganda [53], Nigeria [5], and India [50]. This could be explained as the prevalence of CPD is high in Ethiopia, where girls are small in stature, grow up malnourished, marry at a young age, and become pregnant before their pelvis is fully grown [60]. Additionally, a cross-sectional study of obstetric fistula patients in Ethiopia revealed that the mean ages at the first marriage and at the delivery that caused the fistula were 14.7 and 17.8 years respectively [61]. Indeed, 13% of the girls surveyed in the study in 2016 between 15 and 19 years of age had begun childbearing, including 1.6% of 15 year-olds, 4.4% of 16-year-olds and 13% of 17 year-olds [62]. Malposition and malpresentation were also responsible for 27.4% of obstructed labor, which was consistent with a study conducted at Pakistan Public Sector University [63].

Sepsis was the commonest maternal complication of obstructed labor accounted for 38.08% of cases. The result is in line with studies conducted in Uganda [53], India [50], Eastern Nigeria [52], and the United States [64]. Additionally, postpartum hemorrhage resulted in 33.54% of obstructed cases. The result is supported by studies in Norway [65] and Le Ray et al. [66].

Uterine rupture resulted in 29.84% of obstructed cases. The result is supported by the study conducted in Uganda [67], Dar es Salaam, and the USA [68]. The reason for this could be during obstructed labor there is an impossible barrier (obstruction) preventing its descent despite strong uterine contractions, which increases the risk of uterine rupture.

This review revealed that obstructed labor results stillbirth 38.59% of cases. The result is in line with studies in Boston, Massachusetts, United States [64], and Pakistan [51]. The possible reason might be obstructed labor is when the baby does not exit the pelvis during childbirth due to being physically blocked, despite the uterus contracting, resulted in the baby not getting enough oxygen which may result in death. Moreover, as labor is obstructed, the fetal head impacts on the soft tissue of the pelvic floor, pinning the bladder base and the urethra against the pelvic bone. In the absence of any intervention, this condition may last for several days; the fetus may die and the results stillbirth.

Maternal death has also resulted in 17.27% obstructed labor cases in Ethiopia. The result is supported by a systematic review in Sub-Saharan Africa [69], Uganda [53], and Eastern Nigeria [52]. This shocking figure is certainly an underestimation of the problems, because deaths due to obstructed labor are often classified under other complications (such as sepsis, postpartum hemorrhage or ruptured uterus). This could be explained by obstructed labor results, dehydration, exhaustion, fistula, uterine rupture, sepsis, postpartum hemorrhage, anemia, and shock which all could result in maternal death.

### Limitation

Since it is the first systematic review and meta-analysis, it is taken as a strength. The included articles were restricted to the English language only; this is a limitation of the study as it missed studies published in local languages. Additionally, one of the limitations of this systematic review is the credibility of the unpublished and non-peer-reviewed publications included in this review.

## Conclusion

This study revealed the high incidence of obstructed labor and its complications in Ethiopia. Not having antenatal care follow-up, rural residency, and referred from health centers and visited hospitals after at least 12 h of labor were contributing factors for the incidence of obstructed labor. Additionally, the major causes of obstructed labor were cephalo-pelvic disproportion and malpresentation and malposition. Sepsis, stillbirth, postpartum hemorrhage, uterine rupture, and maternal death were the commonest complications of obstructed labor among mothers who gave birth in Ethiopia. Therefore, to prevent the incidence of obstructed labor; promoting ANC service utilization during pregnancy, improving the referral system, and infrastructure to reach health faculty that had a capacity to manage obstructed labor is recommended. Moreover, it is better to promote institutional service utilization for the prevention and early management of obstructed labor and its complications.

## Supplementary Information

**Additional file 1. **Prisma checklist.

**Additional file 2. **NOS quality assessment score for the included studies.

## Data Availability

The data sets generated during the current study are available from the corresponding author on reasonable request.

## Abbreviations

AA: Addis Ababa
CI: Confidence Interval
CPD: Cepalo-Pelvic Disproportion
AOR: Adjusted Odds Ratio
PRISMA: Preferred Reporting Items for Systematic Reviews and Meta-Analyses
SNNPR: Southern Nation Nationality and Peoples Representative
