# Supplementary material for: Incidence, causes, and maternofetal outcomes of obstructed labor in Ethiopia: systematic review and meta-analysis
Source: Reprod Health. 2021 Mar 10;18:61. doi: 10.1186/s12978-021-01103-0 (PMC7944638; doi:10.1186/s12978-021-01103-0)
Supplement: Supplementary file 2 — Additional file 2. NOS quality assessment score for the included studies. [file 12978_2021_1103_MOESM2_ESM.docx]

The Newcastle-Ottawa Scale (NOS) for assessing the quality of included studies in this systematic review and meta-analyses

| **NOS scale for cross sectional studies** | **Selection** | | | | **Comparability** | **Outcome** | | | **Total score** |
| --- | --- | --- | --- | --- | --- | --- | --- | --- | --- |
|  | Representativeness  (1) | Sample size  (1) | Non-respondents  (1) | Ascertainment of the exposure (risk factor)  (2) | The subjects in different outcome groups are comparable, based on the study design or analysis. Confounding factors are controlled (2) | Assessment of the outcome  (2) | Statistical test  (1) | |  |
| Shimelis F.et al(2010)([35](#_ENREF_35)) | * | * | * | ** | * | ** | * | | 9 |
| Andualem H.et al(2015)([37](#_ENREF_37)) | * | * | * | * | ** | ** | * | | 9 |
| Amanuel G. et al(2001)([21](#_ENREF_21)) | * | * | * | ** | * | ** | * | | 9 |
| Ashebir G. et al(2002)([41](#_ENREF_41)) | * | * | * | * | * | ** | * | | 8 |
| Tizita T. et al (2018)([22](#_ENREF_22)) | * | * | * | ** | * | ** | * | | 9 |
| Sisay S. et al (2017)([39](#_ENREF_39)) | * | * | * | ** | * | ** | * | | 9 |
| Tewodros E. et al (2015)([48](#_ENREF_48)) | * | * | * | ** | * | ** | * | | 9 |
| Daniel S. et al (2019)([14](#_ENREF_14)) | * | * | * | * | * | ** | * | | 8 |
| JOHANNES P. et al(2013)([47](#_ENREF_47)) | * | * | * | ** | * | ** | * | | 9 |
| Asnakech T. et al(2016)([36](#_ENREF_36)) | * | * | * | * | * | ** | * | | 9 |
| Gebresilasea G. et al(2017)([43](#_ENREF_43)) | * | * | * | ** | * | ** | * | | 8 |
| Wayu A. et al(2014)([44](#_ENREF_44)) | * | * | * | ** | * | ** | * | | 9 |
| Oumar S. et al(2016)([45](#_ENREF_45)) | * | * | * | ** | * | ** | * | | 9 |
| **NOS scale for case control studies** | **Selection** | | | | **Comparability** | **exposure** | | | Total score |
|  | Adequacy of case definition | Representativeness | Selection of controls | Definition of controls | Comparability of cases and controls on the basis of the design or analysis | Assessment of the exposure | Same method of ascertainment for cases and controls | Non-Response rate |  |
| Mulugeta D et al(2020)([46](#_ENREF_46)) | * | * | * | * | ** | * | * | * | 9 |
| Ritbano A. et al (2019)([42](#_ENREF_42)) | * | * | * | * | ** | * | * | * | 9 |
| Yemane B. et al(1999)([38](#_ENREF_38)) | * | * | * | * | ** | * | * | * | 9 |
